# E-cadherin loss alters cytoskeletal organization and adhesion in non-malignant breast cells but is insufficient to induce an epithelial-mesenchymal transition

**DOI:** 10.1186/1471-2407-14-552

**Published:** 2014-07-30

**Authors:** Augustine Chen, Henry Beetham, Michael A Black, Rashmi Priya, Bryony J Telford, Joanne Guest, George A R Wiggins, Tanis D Godwin, Alpha S Yap, Parry J Guilford

**Affiliations:** Cancer Genetics Laboratory, Department of Biochemistry, University of Otago, Dunedin, 9054 New Zealand; Division of Molecular Cell Biology, Institute for Molecular Bioscience, The University of Queensland, St Lucia, Brisbane 4072 Australia

**Keywords:** *CDH1*, Cytoskeletal modelling, Adhesion, Migration, EMT

## Abstract

**Background:**

E-cadherin is an adherens junction protein that forms homophilic intercellular contacts in epithelial cells while also interacting with the intracellular cytoskeletal networks. It has roles including establishment and maintenance of cell polarity, differentiation, migration and signalling in cell proliferation pathways. Its downregulation is commonly observed in epithelial tumours and is a hallmark of the epithelial to mesenchymal transition (EMT).

**Methods:**

To improve our understanding of how E-cadherin loss contributes to tumorigenicity, we investigated the impact of its elimination from the non-tumorigenic breast cell line MCF10A. We performed cell-based assays and whole genome RNAseq to characterize an isogenic MCF10A cell line that is devoid of *CDH1* expression due to an engineered homozygous 4 bp deletion in *CDH1* exon 11.

**Results:**

The E-cadherin-deficient line, MCF10A *CDH1-/-* showed subtle morphological changes, weaker cell-substrate adhesion, delayed migration, but retained cell-cell contact, contact growth inhibition and anchorage-dependent growth. Within the cytoskeleton, the apical microtubule network in the *CDH1*-deficient cells lacked the radial pattern of organization present in the MCF10A cells and F-actin formed thicker, more numerous stress fibres in the basal part of the cell. Whole genome RNAseq identified compensatory changes in the genes involved in cell-cell adhesion while genes involved in cell-substrate adhesion, notably *ITGA1, COL8A1*, *COL4A2* and *COL12A1*, were significantly downregulated. Key EMT markers including *CDH2, FN1, VIM* and *VTN* were not upregulated although increased expression of proteolytic matrix metalloprotease and kallikrein genes was observed.

**Conclusions:**

Overall, our results demonstrated that E-cadherin loss alone was insufficient to induce an EMT or enhance transforming potential in the non-tumorigenic MCF10A cells but was associated with broad transcriptional changes associated with tissue remodelling.

**Electronic supplementary material:**

The online version of this article (doi:10.1186/1471-2407-14-552) contains supplementary material, which is available to authorized users.

## Background

E-cadherin, encoded by the tumor suppressor gene *CDH1,* is a homophilic cell-to-cell adhesion protein localized to the adherens junctions of all epithelial cells [1]. Its cytoplasmic domain effectively creates a bridge between the cytoskeletons of adjacent cells by interacting with both cortical actin filaments and the microtubule network [2]. These and other interactions [3] extend E-cadherin’s functionality beyond cell-cell adhesion to roles in establishing and maintaining cell polarity, differentiation, stemness, cell migration and the mediation of signalling through various proliferation and survival pathways including WNT and EGFR [1–5].

Abrogation of *CDH1* expression by mutation, deletion or promoter hypermethylation is a feature of many epithelial tumours, including prostate, ovarian, lung and hepatocellular carcinomas, and is the hallmark of both the sporadic and familial forms of diffuse gastric cancer (DGC) and lobular breast cancer (LBC) [1, 6]. In both LBC and DGC, *CDH1* inactivation can be an early initiating event [7, 8], whereas in other tumour types including prostate, lung, ovarian and colon, its downregulation is usually considered to be a late event that promotes an increase in invasive capacity [9]. Increased invasiveness following *CDH1* downregulation is related, at least in part, to the central role played by E-cadherin in the de-differentiation process known as the epithelial-mesenchymal transition (EMT) [10]. During the EMT, epithelial cells lose polarity and normal cell-cell adhesion, acquiring a mesenchymal phenotype with higher motility and an increase in cell-extracellular matrix (ECM) connections [9, 11]. The EMT is associated not only with increased tumor invasion and metastasis, but also poor outcome, drug resistance and an increase in the number of cancer stem-like cells [9, 12]. E-cadherin downregulation has been shown to be sufficient to induce an EMT in some [4, 9, 10, 13], but not all [14, 15], cancer cell lines/models. However, it remains unclear whether its loss can induce an EMT in cells which have not already undergone malignant transformation [16].

Clues to the influence E-cadherin loss has on tumorigenesis and the initiation of the EMT come from study of the multifocal gastric signet ring cell carcinomas (SRCCs) that occur in Hereditary Diffuse Gastric Cancer (HDGC) families. HDGC is a familial cancer syndrome caused by germline mutation of the *CDH1* gene and is typified by highly penetrant DGC and an elevated risk of LBC [17]. With few exceptions, mutation carriers develop tens to hundreds of gastric foci of SRCC, sometimes with enrichment in the transition zone between the antrum and body [18]. LBC and lobular carcinoma *in situ* (LCIS) are also observed to be multifocal in female mutation carriers (V.Blair, pers. comm). The multifocal gastric SRCCs are E-cadherin-negative and almost exclusively stage T1a tumours confined to the *lamina propria*. Lineage markers suggest that the foci develop from mucous neck cells that have invaded through the basement membrane of the gastric gland [19]. Invasion is likely to be triggered by inactivation of the wild-type *CDH1* allele through mechanisms including promoter hypermethylation [6]. In one model [20], E-cadherin loss creates instability in the orientation of the mitotic spindle, leading to a proportion of the cell divisions occurring out of the epithelial plane with subsequent displacement of daughter cells into the *lamina propria*. The multifocal SRCC foci in the gastric mucosa are known to be relatively indolent, but show unpredictable progression to advanced disease. A small percentage of foci show characteristics of an EMT, and this change is associated with tumour progression [19]. However, the absence of an EMT-like phenotype from the majority of SRCC foci suggests that E-cadherin loss alone is insufficient to induce an EMT in this relatively normal genetic background.

MCF10A is a spontaneously immortalized, non-transformed mammary epithelial cell line derived from human fibrocystic tissue. Although it does carry cytogenetic abnormalities associated with *in vitro* cultured mammary epithelial cells, including *p16* and *p14ARF* deletion and *MYC* amplification [21], MCF10A is considered a “normal” breast epithelial cell due to its near diploid, stable karyotype and characteristics of normal breast epithelium such as lack of tumorigenicity in nude mice, lack of anchorage-independent growth [22] and ability to form mammospheres in culture [21]. Here we have used cell-based assays and whole genome RNAseq to characterize an isogenic MCF10A cell line that is devoid of *CDH1* expression due to an engineered homozygous 4 bp deletion in *CDH1* exon 11. We show that E-cadherin loss disrupts the organization of the cell’s actin and microtubule cytoskeletons and modifies its adherence and migration characteristics but is insufficient to induce an EMT.

## Methods

### Cell culture

MCF10A cells (product no: CRL 10317), a non tumorigenic mammary epithelial cell line, and the derived isogenic line with *CDH1* knock out (MCF10A *CDH1*-/-) using CompoZr ZFN technology (product no: CLLS1042) were purchased from Sigma. The MCF10A isogenic lines were cultured in DMEM/F12: (1:1) (Invitrogen) with 5% horse serum (Invitrogen), 10 μg/ml Actrapid Penfil neutral insulin (Novo Nordisk Pharmaceuticals Ltd), 20 ng/ml human epidermal growth factor (Peprotech), 100 ng/ml cholera toxin, and 500 ng/ml hydrocortisone (Sigma) [21]. Cells were grown at 37°C with 5% CO_2_, seeded into T75 flasks at densities of 3.0 × 10^5^ and 4.5 × 10^5^, respectively and passaged at 90% confluency (~3 days) for a maximum of ten passages (http://brugge.med.harvard.edu/protocols).

### Western blot

MCF10A and MCF10A *CDH1*-/- cells were grown for 72 h to 90% confluency in T25 flasks and lysed using cell culture lysis reagent (Promega) containing cOmplete mini protease inhibitor (Roche). BCA assays (Thermo) were performed to equalize total protein loaded. Proteins were separated on 10% SDS-PAGE gel for 2 h, followed by blot transfer at 100 V for 1 h. Immunoblotting was performed using rabbit anti-E-cadherin antibody (Santa Cruz, SC7870) at 1:200 dilution overnight, or rabbit anti-α-actin primary antibody (Sigma) at 1:1,500 dilution overnight followed by anti-rabbit HRP-linked secondary antibody (Santa Cruz) at 1:5,000 dilution for 1 h. Chemiluminescence was performed using Pierce ECLplus reagent (Thermo) and imaged using LAS-3000 (Fujifilm).

### Immunofluorescence

MCF10A and MCF10A *CDH1-/-* cells were seeded on Coverglass slides (Labtek) and grown to confluence for 72 h. Cells were fixed with 4% paraformaldehyde then permeabilized with 0.2% Triton-X100 in PBS for 5 min at room temperature. Cells were blocked with 10% FBS in PBS for 1 h at room temperature. E-cadherin primary antibody (Santa Cruz, SC-7870) used at 1:250. Anti-rabbit secondary antibody conjugated with AlexaFluor-488 (Invitrogen) used at 1:750. Immunofluorescence images were acquired with an Olympus IX71 microscope, under 40× objective.

### Proliferation assay

MCF10A and MCF10A *CDH1*-/- cells were seeded at densities of 2.0 × 10^3^ and 4.0 × 10^3^ in three replicates in 96 well E-plates and incubated at 37°C in 5% CO_2_. The growth rate was monitored in real time at 15 min intervals for 96 h using the xCELLigence platform (Roche). Both cell lines were also seeded at the same densities into 96 well flat clear bottom black plates (Corning) and grown at 37°C in 5% CO_2_ and imaged every 2 h for 96 h using the IncuCyte 2011A FLR (Essen Bioscience). Confluency was determined using the IncuCyte software Confluence v1.5.

### Cell adhesion assay

Cell adhesion assays were performed using the IncuCyte 2011A FLR. MCF10A and MCF10A *CDH1*-/- cells were seeded in six replicates at 2.0 × 10^4^ cells per well in 96 well flat clear bottom plates (Greiner Bio-one) with different surface coatings: no coating for the uncoated, 2 μg/ml collagen (Sigma), 2 μg/ml fibronectin (BD Bioscience), 8 μg/ml vitronectin (Invitrogen), 8 μg/ml laminin (Invitrogen) and grown at 37°C, 5% CO_2_. Images were acquired every 2 h for 8 h using the automated image acquisition software. Cell numbers at each time point were also determined using the Cell Counter plugin (http://rsbweb.nih.gov/ij/plugins/cell-counter.html) in ImageJ [23].

### Scratch wound assay

Scratch wound assay was performed using the IncuCyte 2011A FLR (Essen Bioscience). Briefly, MCF10A and MCF10A *CDH1*-/- cells were seeded in six replicates at densities of 2.5 × 10^4^ and 3.5 × 10^4^ cells per well, respectively, in 96 well Essen ImageLock Plate (Essen Bioscience) with different coating surfaces: no coating for the uncoated condition, 2 μg/ml collagen, 2 μg/ml fibronectin, 8 μg/ml vitronectin, 8 μg/ml laminin. Cells were incubated at 37°C and 5% CO_2_ and grown to 100% confluency. The usage of the Essen imageLock plates ensures wounds are automatically located and registered by the IncuCyte software and analyzed using wound confluence metrics. Precise and reproducible wounds were generated using the 96 PTFE pin WoundMaker (Essen Bioscience) on the confluent monolayer and cells returned to the incubator where images of cells were acquired every 1 h for 35 h under phase contrast microscopy. Wound confluence was graphed over time to quantitatively evaluate the characteristic of wound closing using the IncuCyte software, Wound Confluence v1.5.

### Soft agar assay

An overlay of 2.0 × 10^4^ MCF10A and MCF10A *CDH1*-/- cells and 2.0 × 10^3^ MCF7 cells in 0.35% agar in medium were plated over a base layer of 0.5% agar (Applichem) and grown at 37°C with 5% CO_2_. Growth medium was added the next day and replenished twice a week. After 24 days, growth medium was removed and MTT (Sigma) solution was added (final concentration 2 mg/ml), and the plates further incubated at room temperature for 4 h with gentle shaking. The MTT solution was then removed and washed. Images were taken using Image Scanner 3 and colonies counted. The experiment was performed with at least two technical replicates for each cell line.

### Immunofluorescence confocal microscopy

MCF10A and MCF10A *CDH1-/-* cells were seeded on glass coverslips coated with fibronectin (Becton Dickinson) and allowed to grow to confluence for 48-72 h. Cells were fixed with ice-cold methanol for 5 min on ice for microtubule staining or fixed with 4% paraformaldehyde in cytoskeleton stabilization buffer (10 mM PIPES pH 6.8, 100 mM KCl, 300 mM sucrose, 2 mM EGTA, 2 mM MgCl2) on ice for 20 min and then permeabilized with 0.25% Triton-X100 in PBS for 5 min at room temperature for F-actin staining. Cells were blocked with 5% milk in PBS for 1 h at room temperature. Primary antibodies used: mouse monoclonal antibody (mAb) directed against the ectodomain of E-cadherin (HECD-1) (a gift from Peggy Wheelock, University of Nebraska, Omaha, NE; with the permission of M. Takeichi) 1:50; rabbit polyclonal Ab (pAB) against E-cadherin (generated in-house) [24] 1:1000; rat monoclonal [YOL1/34] antibody against tubulin (Abcam, # ab6161); 1:100 rabbit polyclonal antibody against ZO-1 (Invitrogen, # 61-7300). F-actin was stained with AlexaFluor 488-phalloidin, 1:500 (Invitrogen). Secondary antibodies were species-specific antibodies conjugated with AlexaFluor-488, -594 or -647 (Invitrogen) for immunofluorescence (1:500). For immunofluorescence, confocal images were acquired with a Zeiss 710 Meta laser scanning confocal microscope, with a 60× objective, 1.4 NA oil Plan Apochromat immersion lens with 0.6-1.0 μm optical sections. Contrast adjustment and z-projections of raw images were done using ImageJ software (National Institutes of Health) [23] and Illustrator (Adobe).

### RNASeq

MCF10A and *MCF10A CDH1-/-* cells were seeded at densities of 2.0 × 10^5^ and 3.5 × 10^5^ cells respectively in duplicate in a six well dish and grown until 70% confluency, with a medium change at 24 h. Total RNA was extracted at 48 h post seeding using quick-RNA Miniprep Kit (Zymo) according to the manufacturer’s protocol. RNA yield and purity were assessed using Qubit (Invitrogen) and the Agilent 2100 Bioanalyser. cDNA library preparation was performed by New Zealand Genomics Limited using Illumina TruSeq RNA preparation version 2.0. Each library had inserts of 200 bp and sequence reads were generated from one lane of an Illumina HiSeq™ 2000 run. Bowtie2 and Cufflinks version 2.0.1 software packages were used to align the read data to human genome build GRC37 and annotated with BiomaRt using Ensembl dataset ”hsapiens_gene-_ensembl”. Unannotated genes were removed and remaining count data was normalized using EdgeR [25]. The per gene read counts were imported into the statistical software package R (http://www.r-project.org), and analyzed using the functionality included in the edgeR and limma packages. Briefly, TMM (trimmed mean of M values) normalization was applied to generate normalized count data, and the lmFit command was used to fit a linear model to the data for each gene. Normalized data were converted to log-cpm (counts per million reads) prior to analysis using the voom command in limma. Differential expression results for MCF10A *CDH1-/-* vs MCF10A were written to CSV files, viewable in Excel (limma moderated t- statistic produced for each comparison, per gene, with FDR p value adjustment applied). Gene Ontology (GO) functional enrichment analysis was carried out using GATHER [26].

## Results

### Characterization of MCF10A CDH1-/- appearance and growth characteristics

**Figure 1:**
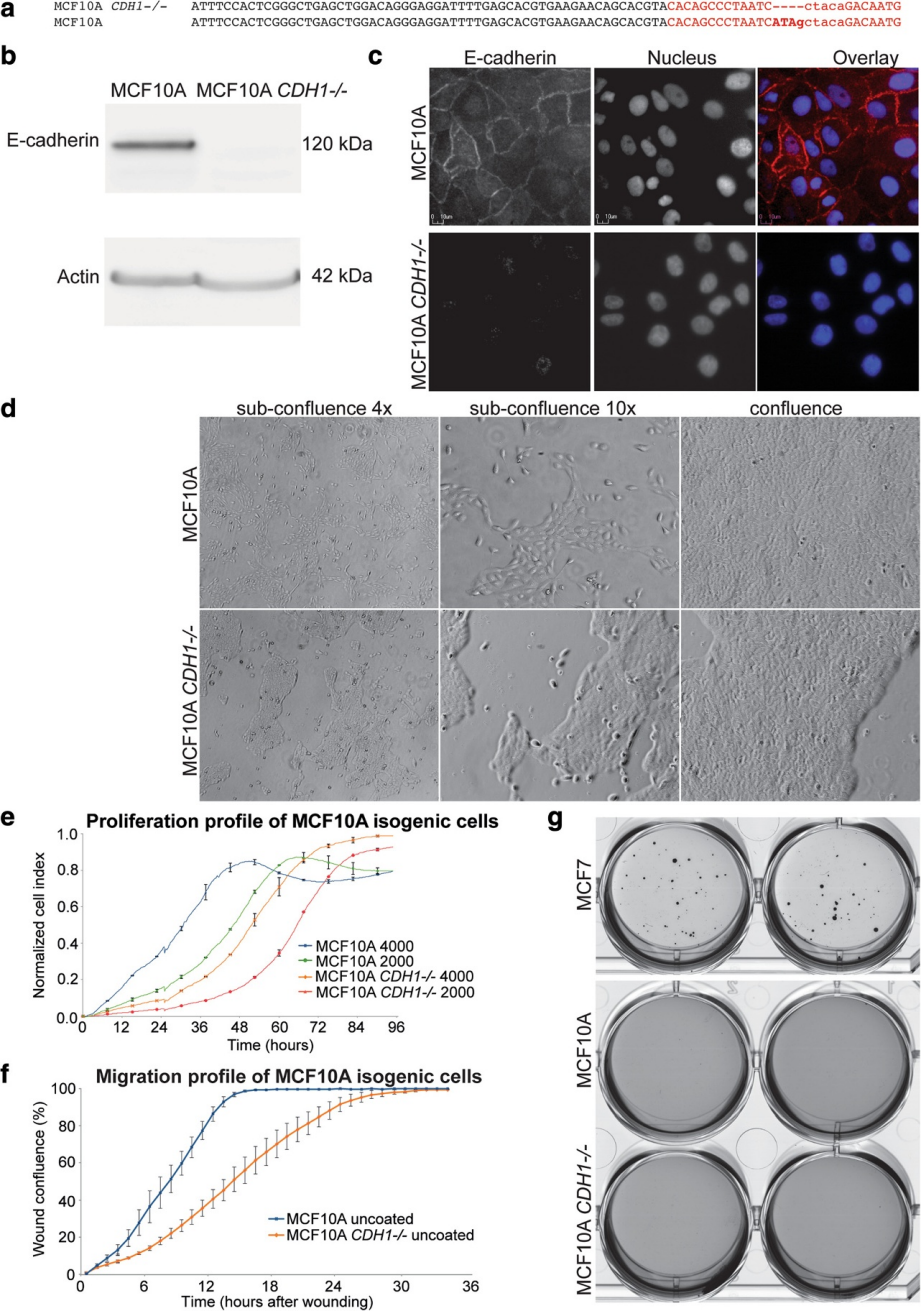
**Characterization of MCF10A** ***CDH1-/-*** **cells. a)** *CDH1* sequence from MCF10A *CDH1-/-* and wildtype MCF10A cell lines depicting the engineered 4 bp deletions as determined by RNAseq. The specified deletion was attributed to ZFN editing on exon 11 of both *CDH1* alleles in MCF10A *CDH1-/-* cells. The ZFN binding site is represented by bases in red uppercase and the ZFN cut site is represented in red lowercase. **b)** Immunoblot of MCF10A *CDH1-/-* confirming the loss of E-cadherin expression as a result of the 4 bp deletion with α-actin as loading control. The cropped images are a composite of the same nitrocellulose immunobloted with antibodies against E-cadherin followed by α-actin (Additional file 1: Figure S1). **c)** Immunofluorescence showing loss of E-cadherin from the cell junctions in MCF10A *CDH1-/-* but not wildtype MCF10A cells. **d)** Comparison of growth morphology between MCF10A *CDH1-/-* and wildtype at subconfluence and full confluence. At subconfluence, MCF10A *CDH1-/-* showed clustered and contracted distribution while some wiltdtype MCF10A cells exhibited more mesenchymal morphology. At full confluence, both isogenic cells retained epithelial cobblestone-like morphology, although MCF10A *CDH1-/-* displayed gaps not observed in wildtype cells. **e)** Comparing cell proliferation profile between both MCF10A isogenic cells. A measure of cell proliferation was represented by the normalized cell index taken from impedence measurements generated by cells grown over 96 h on a 96-well E-Plate on the xCELLigence. **f)** The time course of cell migration was quantified using IncuCyte wound confluence at 1 h intervals over 35 h. MCF10A *CDH1-/-* cells were shown to take significantly longer in wound closing compared to wildtype cells. **g)** Soft agar assay to determine anchorage-independent growth as a result of E-cadherin loss. Anchorage-independent growth was observed only in the positive control MCF-7 cells, but not in either of the MCF10A isogenic cells. Representative images from one of two biological replicates were presented.

A MCF10A *CDH1*-/- cell line carrying a homozygous 4 bp deletion in exon 11 of *CDH1* has recently been developed using zinc finger nuclease (ZFN) technology (Sigma-Aldrich, Saint-Louis). The 4 bp deletion (Figure 1a) at mRNA position 1820-1823 results in a frameshift predicted to give rise to a premature termination codon at position 1868, generating a truncated protein of 582 amino acids that lacks the extracellular cadherin repeat 4 domain, transmembrane region and cytoplasmic domain. Immunoblotting and immunofluorescence confirmed the absence of E-cadherin expression from the MCF10A *CDH1*-/- line (Figure 1b-c; Additional file 1: Figure S1). Subconfluent MCF10A *CDH1*-/- cells exhibited a more rounded morphology and grew in a clustered and contracted distribution distinct from the wildtype MCF10A which had a more elongated morphology (Figure 1d). At full confluence, both MCF10A and MCF10A *CDH1*-/- cells retained cobblestone morphology typical of epithelial cell lines. The MCF10A *CDH1*-/- cells however, presented gaps, unlike the even monolayer distribution observed in wildtype MCF10A (Figure 1d). Both the MCF10A and the MCF10A *CDH1-/-* cells maintained normal contact growth inhibition (Figure 1d).

To estimate the effect of E-cadherin loss on the growth kinetics of the MCF10A cells, the isogenic pair of cell lines were seeded at a density of 2.0 × 10^3^ and 4.0 × 10^3^ cells per well in 96 well E-plates and growth followed using the xCELLigence real time system (Roche, Basel). The MCF10A *CDH1*-/- cells showed a prolonged lag phase when compared to the wildtype MCF10A cells (Figure 1e). However, once both cell lines achieved log phase growth, the doubling time of the two lines was almost identical with MCF10A being only slightly shorter (13 h) compared to the MCF10A *CDH1*-/- cells (14 h) (Figure 1e).

We also performed scratch wound assays to measure cell migration in real-time over 35 h post wounding. Wound closure was quantitatively evaluated over time using Wound Confluence v1.5 (time point t = 0 h, corresponds to 2.5 h post wound generation). MCF10A *CDH1*-/- cells migrated markedly slower and took longer to close the wound (t = 27.4 ± 4.1 h) compared to wildtype cells (t = 15.0 ± 1.6 h) (Figure 1f).

Finally, the number of nucleoli present per cell were noticeably reduced in MCF10A *CDH1-/-* compared to wildtype: 87% of MCF10A *CDH1*-/- cells have one or two nucleoli per cell compared to 42% of MCF10A cells (Additional file 2: Figure S2). The majority of wildtype MCF10A cells (57%) had three or more nucleoli compared to 12% of MCF10A *CDH1*-/- cells (Additional file 2: Figure S2). The reduction in nucleoli number is suggestive of a decreased demand or capability for ribosome biogenesis.

### Loss of CDH1 does not enable anchorage-independent growth

To determine if E-cadherin loss would cause MCF10A cells to become tumorigenic, we performed soft agar colony formation assays to monitor anchorage–independent growth. After 24 days in soft agar, no colonies were formed for MCF10A cells, consistent with a previous study [22]. Likewise, MCF10A *CDH1-/-* cells did not show any colony growth (Figure 1g). This result showed neither MCF10A nor MCF10A *CDH1-/-* cells exhibit the ability to divide and proliferate in the absence of adhesion to the substratum.

### MCF10A CDH1-/- cells show altered actin and tubulin cytoskeletal arrangement

**Figure 2:**
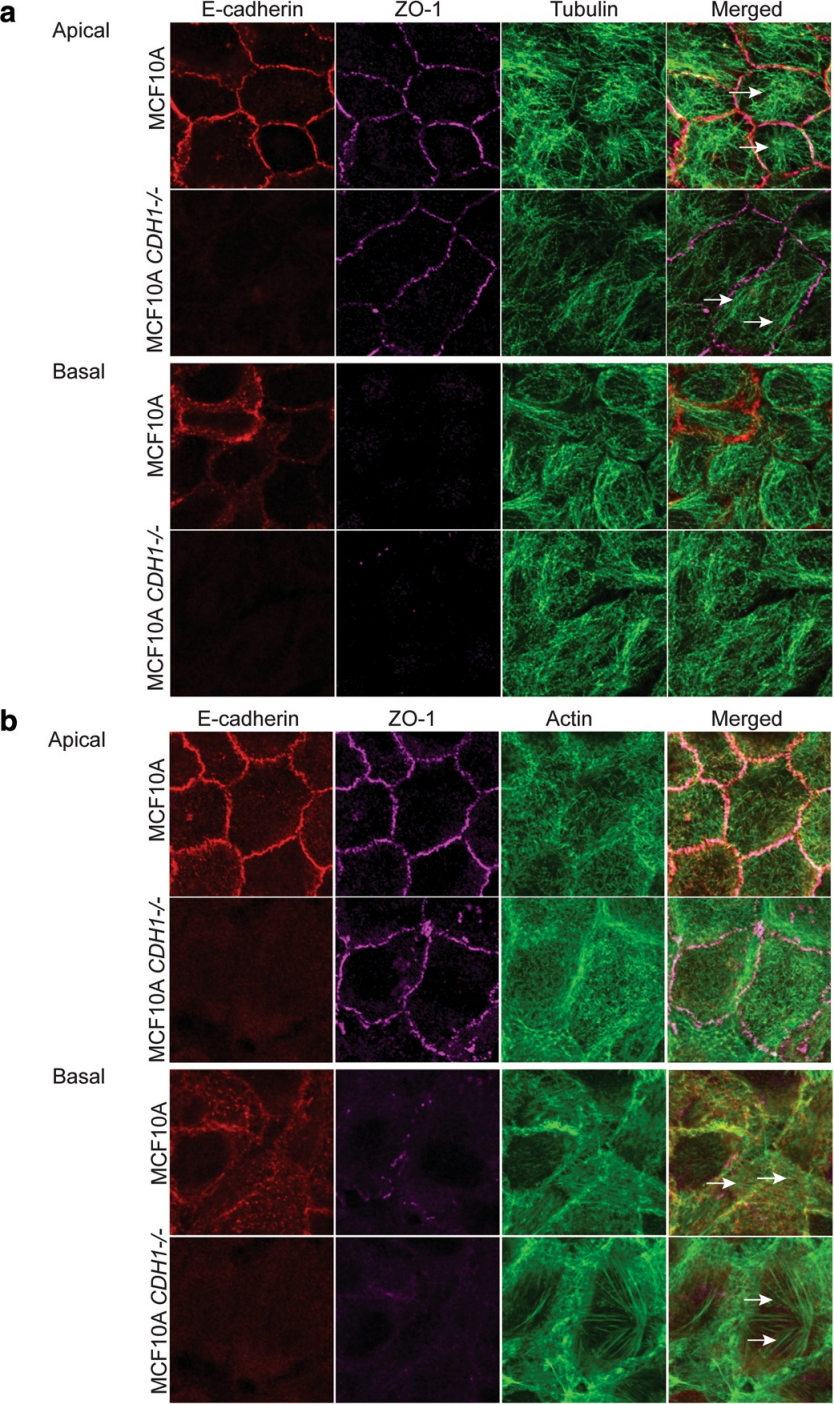
**E-cadherin loss altered cytoskeletal organization in MCF10A** ***CDH1-/-*** **cells. a)** Loss of E-cadherin altered tubulin cytoskeletal arrangement. On the apical surface of MCF10A cells, the microtubules showed radial pattern of organization (indicated by white arrows) with the minus end densely anchored in the centre and the plus end extending towards the cell cortex. However, in MCF10A *CDH1 -/-* cells had gross defect in the radial pattern of organization and often oriented parallel to the cortex (indicated by white arrows). At the basal surface, the microtubules form a meshwork like structure with no striking difference observed between the two cell lines. **b)** Loss of E-cadherin altered actin cytoskeletal arrangement. On the apical surface of MCF10A cells, actin forms cross-linking filamentous meshwork while basally it organizes itself into stress fibres like structure. Overall apical actin meshwork looks similar in both MCF10A isogenic cells but basally there are more and thicker stress fibres in MCF10A *CDH1-/-* cells (indicated by white arrows).

To directly observe the effects of E-cadherin knock-out on the cytoskeleton of MCF10A cells, we examined the microtubule and actin cytoskeletons using immunofluorescence staining for α-tubulin and F-actin. On the apical surface of MCF10A cells, the microtubules displayed a prominent radial pattern of organization with minus ends anchored densely in the centre and the plus ends extending towards the cell cortex (Figure 2a). However in MCF10A *CDH1*-/- cells, the microtubules were less dense and there was a gross defect in the radial pattern of organization, often oriented parallel to the cell cortex (Figure 2a). At the basal surface of the cells, the microtubules formed a meshwork-like structure and no striking differences in organization between MCF10A and MCF10A *CDH1*-/- cells were observed. Apically in MCF10A cells, actin forms a cross-linking filamentous meshwork while basally it organizes itself into stress fibre like structures (Figure 2b). The apical actin meshwork looked similar in MCF10A *CDH1-/-* cells, but basally the stress fibres were thicker and more numerous in the E-cadherin-deficient cells.

### Loss of CDH1 impacts on the transcription of diverse cell-cell adhesion genes

To elucidate the impact of E-cadherin loss at the transcriptional level, we performed genome-wide RNAseq on the MCF10A and MCF10A *CDH1*-/- cells. An average of 6.55 × 10^7^ reads were obtained per library. Using a cut off of +/- log_2_1.00 and an adjusted p value of <0.05, a total of 1,388 genes were observed to be differentially regulated. Relative to the *CDH1*-expressing cells, 715 genes showed significantly increased expression in the *CDH1*-/- cells and 673 genes had significantly reduced expression. The GO terms [26] most strongly associated with the 1,388 genes included morphogenesis (Bayes factor 34.1), organogenesis (Bayes factor 31.5), development (Bayes factor 30.3), cellular metabolism (Bayes factor 27.8), cell communication (Bayes factor 24.0), cell adhesion (Bayes factor 21.3) and histogenesis (Bayes factor 18.5) (Additional file 3: Table S1).

### Loss of CDH1 expression was associated with expression changes in other cell-cell adhesion genes

Four other cadherins showed significant changes in expression: *CDH2* and *CDH4* were downregulated in the *CDH1*-/- cells by >2 fold, whereas *CDH3* and *CDH16* were upregulated by up to 3.8 fold. Three of the four nectin genes which encode proteins involved in adhesion at the adherens junction were also markedly upregulated by up to 2.2 fold, most notably *PVRL4*. Genes encoding adherens junction-associated proteins (*e.*g. *CTNNB1*) showed little or no change in expression in the MCF10A *CDH1*-/- cells (Additional file 4: Table S2). Ten established tight junction genes showed significant upregulation with five demonstrating upregulation of >2 fold namely, *CLDN1, CLDN4*, *CLDN7*, *OCLN* and *CGN* (Table 1). Two further tight junction genes showed significant downregulation (*JAM3* 1.3 fold decrease, p = 0.004 and *CLDN15* 2.0 fold decrease, p = 0.015), three others had insignificant changes (*TJP1* 1.2 fold decrease, p = 0.154; *TJP2* 1.1 fold increase, p = 0.054 and *CLDN22* 1.1 fold, p = 0.74), while 17 showed negligible expression in both cell lines (Additional file 4: Table S2). Similarly, eight of the eleven expressed desmosome genes and six of the eight expressed gap junction genes also demonstrated increased mRNA expression, most notably *DSG4*, *DSC2*, *JUP*, *GJA5*, *GJB2* and *GJB4,* with fold changes up to 3.9 fold (Table 1). Taken together, this transcriptional data demonstrates that the loss of *CDH1* from the adherens junction is associated with an increase in the expression of genes encoding various tight junction, desmosome and gap junction proteins. This increased expression of other cell-to-cell adhesion genes may partially compensate for the loss of E-cadherin and explains the retention of cell-cell contact and cobblestone epithelial morphology observed at full confluency (Figure 1d).

**Table 1:** **Expression profile of cell adhesion genes in MCF10A** ***CDH1-/-***

Cell-cell adhesion genes			Cell-ECM adhesion genes			Focal adhesion genes		
Gene	FC	Adj. p value	Gene	FC	Adj. p value	Gene	FC	Adj. p value
*CLDN1*	3.1	3.45E-05	*COL2A1*	5.3	3.22E-04	*TLN1*	-1.5	1.48E-02
*CLDN4*	3.8	4.87E-05	*COL4A1*	-2.6	3.45E-05	*TLN2*	-2.0	5.72E-03
*CLDN7*	2.7	1.51E-05	*COL4A2*	-2.5	4.88E-05	*TNS1*	-5.5	7.34E-05
*OCLN*	2.9	2.27E-05	*COL4A4*	-2.9	2.04E-02	*TNS3*	-2.3	2.77E-04
*CRB3*	2.1	5.38E-04	*COL5A3*	3.6	1.07E-03	*UTRN*	-2.4	1.13E-02
*CGN*	3.0	1.80E-04	*COL6A3*	-3.5	2.86E-03	*DLC1*	-2.2	1.19E-03
*(CDH1)*	-10.0	9.10E-06	*COL7A1*	2.0	3.11E-03	*ACTN1*	1.0	4.98E-01
*CDH2*	-2.2	2.37E-04	*COL8A1*	-6.3	1.12E-05	*ACTN4*	1.1	3.03E-01
*CDH3*	1.8	1.51E-05	*COL12A1*	-4.3	4.48E-04	*VCL*	-1.1	4.20E-01
*CDH4*	-2.9	7.79E-04	*COL13A1*	6.1	1.07E-04	*ACTB*	1.2	2.66E-02
*CDH16*	3.8	6.73E-04	*COL18A1*	-2.4	4.07E-04	*ACTG1*	1.3	8.62E-04
*PVRL4*	2.2	1.01E-04	*COL27A1*	-6.3	7.07E-04	*TUBB2A*	1.3	6.73E-03
*DSG4*	3.0	9.73E-04	*COL28A1*	-34.1	4.93E-03	*PTK2*	1.1	1.48E-02
*DSC2*	2.2	2.87E-05	*LAMA1*	-2.5	8.51E-04	*SRC*	-1.3	2.29E-02
*JUP*	2.0	8.71E-06	*LAMA2*	2.3	4.77E-04	*ILK*	1.0	4.20E-01
*GJA5*	2.1	1.13E-02	*FN1*	-7.2	1.78E-04	*RAC1*	1.1	2.29E-02
*GJB2*	3.9	3.88E-06	*ITGA1*	-2.8	6.37E-04	*RHOA*	1.1	1.89E-02
*GJB4*	3.2	7.10E-04	*ITGA10*	2.0	6.92E-04	*ROCK1*	-1.3	4.28E-02
*ICAM1*	-3.6	8.31E-03	*ITGB1*	-1.4	2.91E-04	*PXN*	-1.1	1.65E-02

The *CDH1* transcript level in MCF10A *CDH1-/-* cells was markedly reduced by more than 90% compared to the wild type *CDH1* transcript in MCF10A cells, consistent with nonsense-mediated decay. The table contains a selection of genes involved in cell-cell adhesion, cell-ECM adhesion and focal adhesion including genes with a significant fold change ≥2.0. Other genes with fold change <2.0 are listed in the Additional file 4: Table S2 and Additional file 5: Table S3. FC denotes the fold change relative to MCF10A wildtype expression.

### Loss of CDH1 from MCF10A cells alters expression of the genes involved in cell-ECM adhesion and promotes altered adhesion to basement membrane proteins

In addition to the observed changes in expression of cell-to-cell adhesion genes, the loss of *CDH1* was also associated with significant changes in the expression of cell-substrate adhesion genes (Table 1). Up to 2.8 fold reduction in the expression of the integrin receptor subunit genes *ITGA1, ITGA4, ITGA5, ITGAV, ITGB1, ITGB2* was observed. Only *ITGA10* and *ITGB6* showed significantly increased expression (up to 2 fold; Additional file 5: Table S3). Furthermore, many ECM transcripts demonstrated a marked downregulation in the MCF10A *CDH1*-/- cells compared to the wildtype cells. This was evident for *COL4A1, COL4A2, COL4A4, COL6A3, COL8A1, COL12A1, COL18A1, LAMA1, FN1 and VTN.* Other members of the laminin family *LAMA5, LAMB1, LAMB2 and LAMC1* were also significantly downregulated (Additional file 5: Table S3). Only a small subset of ECM genes were upregulated in MCF10A *CDH1-/-* cells, namely *COL2A1, COL5A3, COL7A1*, *COL13A1 and LAMA2* (Table 1).

Genes encoding focal adhesion components that form linkages between integrins and the actin cytoskeleton were also markedly downregulated (Table 1). *TLN1* and *TLN2,* encoding the talin proteins, are key components in focal adhesion assembly and form linkages between integrins and actin directly or indirectly via vinculin and α-actinin [27]. Both were downregulated by 1.5 fold and 2.0 fold respectively. However, vinculin (*VCL*) and α-actinin (*ACTN1*, *ACTN4*) themselves did not show any significant expression changes. Additionally, genes encoding the tensin proteins which mediate integrin and actin linkages during focal adhesion disassembly [28], *TNS1* and *TNS2* were also significantly downregulated by 5.5 and 2.3 fold respectively. *UTRN* a gene which encodes utrophin, a protein localized to the adherens junction which form linkages to actin [29], was also significantly downregulated by 2.4 fold. In contrast, modest upregulation (up to 1.3 fold) was observed in the genes encoding the actin and microtubule cytoskeletal proteins *ACTB*, *ACTG1* and *TUBB2A* mRNA. While the expression of certain key genes (*SRC*, *PTK2*, *ILK*, *PXN*, *RHOA*) involved in regulating focal adhesion maturation and turnover showed modest expression changes, *DLC1* which encodes a rhoGAP protein that regulates small GTP binding proteins at focal adhesion sites was downregulated by 2.2 fold.

**Figure 3:**
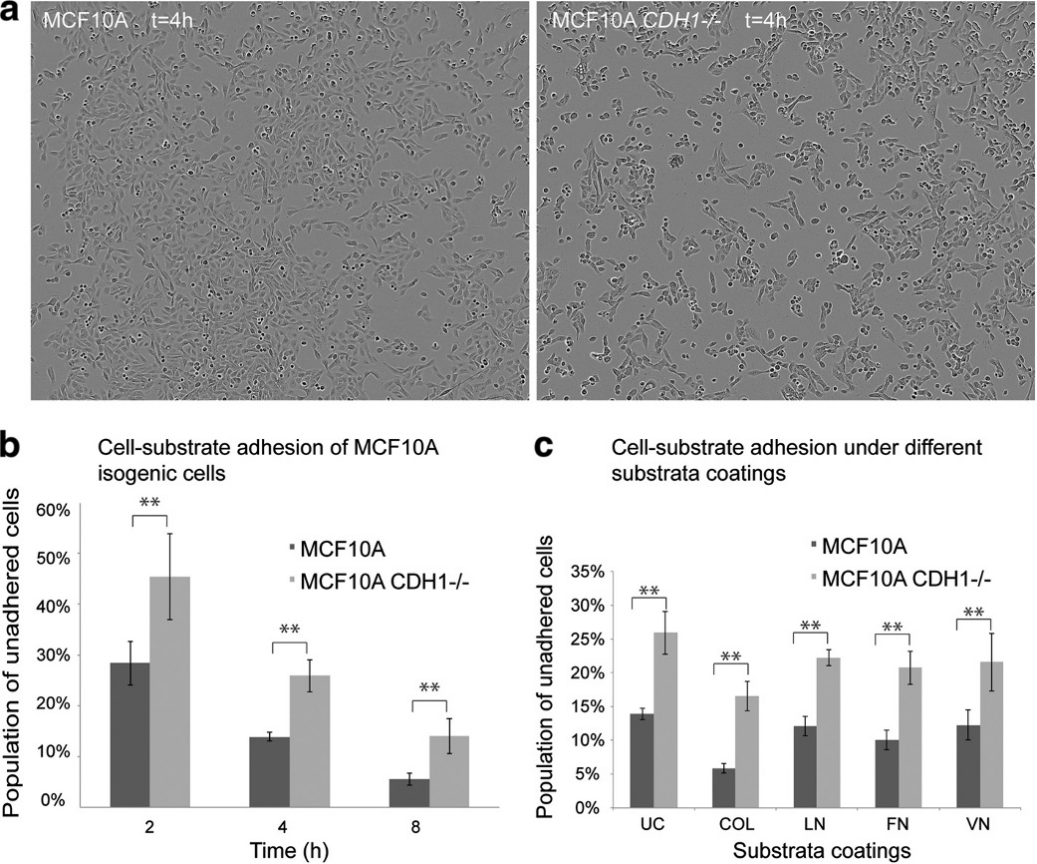
**E-cadherin loss contributes to reduced substrate adhesion in MCF10A** ***CDH1-/-.*** Both isogenic cell lines were seeded at a density of 2.0 × 10^4^ cells and images were taken at 2 h intervals for up to 8 h using the IncuCyte 2011A FLR. **a)** Representative images of cell substrate adherence at 4 h post seeding under uncoated conditions. **b)** Histogram representing the number of adhered to non-adhered cells from both cell lines under uncoated conditions over different time points. Significantly reduced substrate adhesion were observed in MCF10A *CDH1-/-* cells as determined by student t-test p *< 0.01* (represented by **). **c)** Histogram representing the number of adhered to non-adhered cells from both cell lines under different ECM coating conditions (UC, uncoated; COL, collagen; LN, laminin; FN, fibronectin; VN, vitronectin) at 4 h post seeding. For each condition, three images were acquired and the population of non-adhered cells to total cell numbers from each image were counted by two independent researchers and the average taken. Results of experiments done under different ECM coatings showed MCF10A *CDH1-/-* cells consistently showed reduced substrate adhesion compared to the wildtype cells (student t-test; p *< 0.01* represented by **). Furthermore, different ECM coatings provided varying increases in the numbers of adhered cells in both isogenic cells.

**Figure 4:**
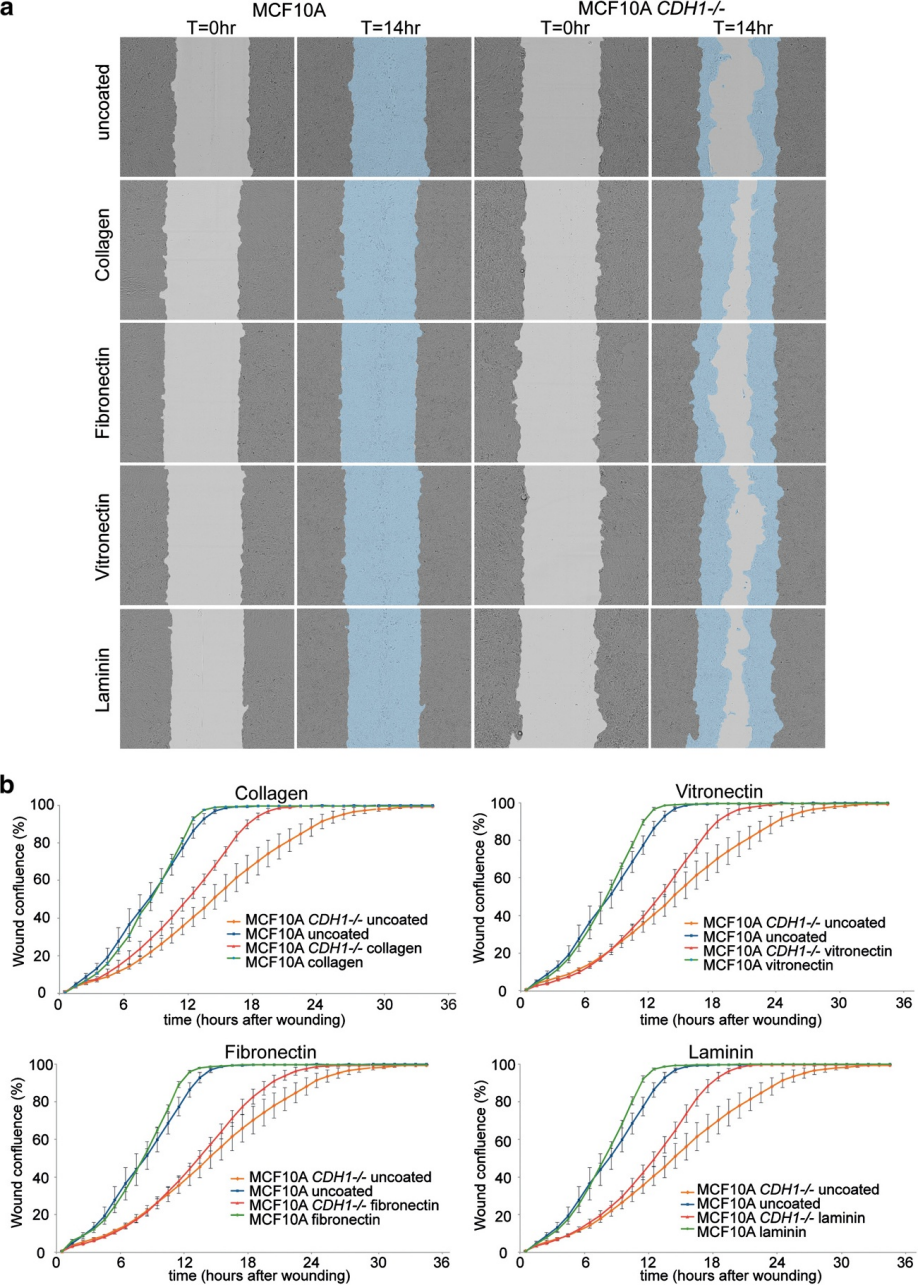
**E-cadherin loss compromises cell migration of MCF10A** ***CDH1-/-.*** Both isogenic cell lines were grown to confluence in complete media, wounds were generated using the 96-well WoundMaker and the data collected over 35 h and analyzed on the IncuCyte. **a)** Representative wounds on MCF10A isogenic cells under different ECM conditions at the start (immediately after wounding) and 14 h post wounding. **b)** The time course of cell migration was quantified using wound confluence at 1 h intervals over 35 h. MCF10A *CDH1-/-* cells were shown to take significantly longer in wound closing compared to wildtype cells under different coating conditions.

To examine this apparent deficit in cell-substrate adhesion in MCF10A *CDH1*-/- cells at the intact cell level, MCF10A and MCF10A *CDH1*-/- cells were plated at an equal density in 96 well uncoated plates and the total number of adherent and non-adherent cells counted after 2, 4 and 8 h. While the proportion of non-adherent cells from both lines decreased with time, their number was significantly higher in MCF10A *CDH1*-/- cells at all three time points (Figure 3a-b) indicating reduced cell-ECM adhesion. This defect in the uncoated substrata might have been due to either decreased matrix secretion and/or decreased integrin expression. To pursue this, we then tested if adhesion could be rescued by pre-coating substrata with matrix proteins. We observed that adhesion to coated substrata is still reduced in E-cadherin deficient cells compared to wildtype MCF10A cells (Figure 3c). The most significant improvement was observed with collagen (p = 0.0006), followed by laminin (p = 0.01), fibronectin (p = 0.04), and vitronectin (p = 0.08). Increased adhesion was also observed in the MCF10A parent cells (Figure 3c) with the most significant gains being with collagen (p = 2.2 × 10^-7^), followed by fibronectin (p = 3.53 × 10^-4^), laminin (p = 0.03), and vitronectin (p = 0.12). While part of the defect seen in Figure 3b may be due to failure of matrix secretion, it does not explain the whole phenomenon which might be due to decreased integrin expression. Coating plates with either collagen, fibronectin, vitronectin or laminin alone also increased MCF10A *CDH1*-/- cell migration as observed in the wound closure assay compared to uncoated wells (Figure 4a-b) but slower compared to wildtype [30]. Gains in migration were less marked in MCF10A wildtype cells (Figure 4b). The increased adhesion and migration of MCF10A *CDH1*-/- cells on ECM protein-coated slides is consistent with the reduced cell-substrate adhesion observed in E-cadherin-negative cells being mediated at least in part by the downregulation of ECM proteins and their associated integrins.

### Loss of CDH1 from MCF10A cells does not induce a coherent change in the expression of EMT-associated genes

The EMT is characterized by the downregulation of epithelial markers-typically including cytokeratins 8, 9 and 18, and various tight junction proteins and the coordinated upregulation of mesenchymal markers including N-cadherin (*CDH2*), vimentin (*VIM*), ECM proteins and matrix metalloproteinases (MMPs). To explore the ability of E-cadherin loss to induce an EMT in isolation of other genetic changes, we mined the relative expression of well characterized EMT genes and their transcriptional regulators in MCF10A and MCF10A *CDH1*-/- cells. None of cytokeratins 8, 9, or 18 were downregulated in the MCF10A *CDH1*-/- cells (Table 2), and as mentioned above, there was a notable trend for tight junction genes such as *CLDN1, OCLN, TJP3,* and *CGN* to be upregulated rather than downregulated (Table 1). Further to the observed trend towards lower expression of ECM genes in the *CDH1*-deficient cells, none of the mesenchymal markers *CDH2, CDH11, VIM, FN1, S100A4, VTN* nor *ACTA2* showed significantly increased expression in the MCF10A *CDH1*-/- cells. Although *S100A4* expression was not significantly changed, 12 other members of the S100A calcium binding protein family (from a total of 16 *S100A*; Additional file 6: Table S4) showed increased expression, including *S100A8* (4.1 fold) and *S100A7* (7.0 fold). The upregulation of transcription factors *SNAI1, SNAI2, TWIST1, TWIST2, ZEB1* and *ZEB2* that are known to promote the EMT and repress *CDH1* expression [9] were themselves unchanged or strongly downregulated in the case of *ZEB2* (Table 2; Additional file 7: Table S5) suggesting the existence of a feedback loop from *CDH1*. Although the overall trend of the epithelial and mesenchymal markers was opposite to that which would occur during an EMT, the MCF10A *CDH1*-/- cells did show a significant increase in the expression of *MMP9* and *14*. This trend towards increased proteolytic activity was supported by the decrease in expression of the metalloprotease inhibitors *TIMP2-3* and the increased expression of 10 members of the kallikrein family of proteases (Additional file 6: Table S4)*.* Taken together, these results do not support E-cadherin loss alone being sufficient to induce a coordinated shift to a mesenchymal phenotype, however they do provide evidence for a direct link between E-cadherin regulation and aspects of tissue remodelling.

**Table 2:** **The relative expression of EMT genes in E-cadherin deficient MCF10A cells**

Epithelial markers			Mesenchymal markers			EMT Transcription factors		
Gene	FC	Adj. p value	Gene	FC	Adj. p value	Gene	FC	Adj. p value
*KRT8*	1.4	7.67E-04	*CDH2*	-2.2	2.37E-04	*SNAI1*	-2.5	1.38E-02
*KRT9*	2.5	2.89E-02	*CDH11*	1.4	3.11E-01	*SNAI2*	1.0	5.83E-01
*KRT18*	1.4	3.86E-04	*VIM*	1.1	6.12E-02	*TWIST1*	-1.1	3.58E-01
*CLDN1*	3.1	3.45E-05	*FN1*	-7.2	1.78E-04	*TWIST2*	-1.2	4.20E-01
*OCLN*	2.9	2.27E-05	*VTN*	-1.3	6.07E-01	*ZEB1*	-1.7	1.23E-02
*TJP3*	1.9	4.69E-04	*CTNNB1*	1.0	4.20E-01	*ZEB2*	-7.3	5.02E-05
*CGN*	3.0	1.80E-04	*ACTA2*	1.0	9.12E-01	*AKT2*	-1.1	8.54E-02
*DSP*	1.0	9.87E-01	*ITGA5*	-1.2	8.75E-03	*MMP1*	5.8	1.45E-02
*SDC1*	1.6	3.11E-05	*ITGAV*	-1.4	2.11E-02	*MMP9*	3.3	9.08E-04

Genes with significant ≥2.0 fold gene expression changes are presented with inclusion of certain key genes. For other ECM genes with fold change <2.0, refer Additional file 5: Table S4. FC denotes the fold change relative to MCF10A wildtype expression.

## Discussion

*CDH1* is widely considered to be an ‘invasion suppressor gene’ whose inactivation is associated with tumor progression. However, the identification of large numbers of early stage gastric and breast cancers in *CDH1* germline mutation carriers [18, 31] demonstrates that E-cadherin loss can also influence the initiation of cancer. To provide a better understanding of the impact of E-cadherin loss in a non-malignant genetic background, we have characterized an E-cadherin-deficient cell line that has been derived from the non-tumorigenic breast line MCF10A using zinc finger nuclease technology.

MCF10A *CDH1-/-* cells largely retained an epithelial cobblestone morphology, although slightly more rounded cells and gaps were observed in the confluent monolayer when compared to wildtype cells. Our transcription analysis suggests that the retention of cell adhesion appears to be associated with compensatory changes in other cell-cell adhesion proteins localized at desmosomes, tight junctions and adherens junctions, including P-cadherin (*CDH3*). Similarly, the targeted loss of E-cadherin from murine skin epithelium is also not associated with a significant loss of cell-cell adhesion, an effect attributed to compensatory upregulation of P-cadherin and desmosomal cadherin in the basal layer [32, 33]. Despite the loss of E-cadherin from the adherens junction, a level of normal cell polarity is retained in the MCF10A *CDH1*-/- cells, based on the apical ZO-1 localization (Figure 2b) and the ability of these cells to form luminal cores in 3D matrigel culture (data not shown). This is consistent with the polarity retention observed in the conditional knockout mouse whereby E-cadherin loss in the epidermis did not alter Par3 and Scribble localization [34]. However, E-cadherin depletion disrupts cell polarization in MDCK cells [35] and can lead to abnormal mitotic spindle orientation in different model systems [20].

The cadherin adhesion complex interacts dynamically with the actin and microtubule cytoskeletons through various multiprotein complexes, allowing mechanosensing, force transmission, vesicle mediated transport of junctional proteins to the zonula adherens, and the regulation of microtubule stability and orientation [2]. *CDH1-*deficient MCF10A cells lacked the radiating microtubule structure observed on the apical side of the wildtype cells (Figure 2a), consistent with a disruption of either plus-end or minus-end microtubule anchoring at the adherens junctions, [36–38]. Not surprisingly, this microtubule reorganization was also associated with downregulation of genes involved in the adherens junction-microtubule axis including *KIFC3,* a gene involved in microtubule minus-ends directed motor found to localize to both the zonula adherens and centrosome and also *NIN* which encode ninein, involved in anchorage to the centrosome.

E-cadherin loss caused no evident difference in the organization of F-actin in the apical region of the MCF10A cells. However, in the basal region there was an increased prominence of stress fibers (Figure 2b) suggesting that basal contractility and traction forces (driven by Rho signalling) might be potentiated. Similar thickening and lengthening of actin stress fibres have been observed in mouse mammary epithelial, NMuMG cells following TGF-β induced EMT [39]. Associated transcriptional changes in the E-cadherin-deficient cells, included upregulation of *RhoA*, *RhoB* and *RhoC,* although their downstream effectors *ROCK1* and *ROCK2* were downregulated.

One striking characteristic of the *CDH1*-deficient MCF10A cells was the reduction in cell-substrate adhesion. Multiple transcriptional changes consistent with this phenotype were observed, including the downregulation of genes encoding ECM proteins [40], integrin subunits and focal adhesion proteins involved in linking integrins with actin stress fibers such as talin, paxillin, tensin and P130Cas. The most marked integrin downregulation was of *ITGA1, ITGA4, ITGA5, ITGAV, ITGB1* and *ITGB2,* genes that encode subunits of the α1β1, α2β1, α3β1, α4β1, α5β1, αvβ1, α1β2 integrin receptors, respectively [27]. The crosstalk between E-cadherin and integrin has been observed previously in major cellular functions including adhesion, migration, proliferation and apoptosis [41]. This is not surprising as both transmembrane adhesion receptors share some common signaling effector molecules, scaffold and cytoskeletal proteins, hence the combined ability to influence coordinated regulation of cell-cell and cell-substrate adhesion crucial in normal cell growth and disease state [10, 41]. The decreased substrate adhesion also translated to reduced cell motility in MCF10A *CDH1-/-* compared to wildtype cells. Another E-cadherin depletion study of MCF10A also showed no gain in cell migration speed [30]. The compromised cell-substrate adhesion and migration could be partially restored when ECM proteins were coated onto the growth surface, however the downregulation of talin (encoded by *TLN1* and *TLN2*) and other genes involved in focal adhesion assembly and disassembly like *TNS1, TSN3* and *UTRN* has probably further compromised traction force [42, 43].

The EMT incorporates a series of coordinated events which involve altered cell-cell and cell-ECM interactions, reorganization of the cytoskeleton and the adoption of a new transcription program to induce and maintain a mesenchymal phenotype. We found little support for E-cadherin loss being able to initiate an EMT in a non-malignant genetic background; deletion of *CDH1* from MCF10A cells was not associated with the reduction in epithelial markers and the coordinated increase in mesenchymal markers such as *CDH2* [44], nor were the EMT regulators *TWIST1*, *TWIST2*, *SNAI1*, *SNAI2*, *ZEB1* and *ZEB2* upregulated [9, 11, 45]. Treatment of MCF10A cells with either TGF-β or FN caused an EMT without downregulation of E-cadherin [39, 46], supporting our observations that *CDH1* loss does not drive the EMT in these cells. A recent study done in a panel of 38 breast cell lines also indicated that E-cadherin loss is not causal for EMT in human breast cancer [15].

The only EMT feature that was clearly activated in the MCF10A *CDH1-/-* cells was increased expression of several metalloprotease genes (*MMP9*, *MMP14*, *MMP15*, *MMP17* and *MMP28*). Increased expression of *MMP2* and *MMP9* has been shown previously in MCF10A cells following TGF-β and ERBB2 induced EMT [47]. We predict that the impact of elevated MMP expression would be detectable in invasion assays using 3D matrices like Matrigel, although such assays were not part of our analysis. In addition, several genes from the S100A calcium-binding protein family, *S100A7*, *S100A8* and *S100A9*, were also strongly upregulated (≥2 fold) in the MCF10A *CDH1-/-* cells. *S100A7*, while not expressed in normal epithelia, is frequently seen to be expressed in pre-invasive ductal carcinoma *in situ* [48] and is associated with EMT.

## Conclusions

In summary, precise deletion of *CDH1* from the non-tumorigenic breast cell line MCF10A does not enable anchorage-independent growth, result in neither enhanced invasiveness *in vitro,* nor lead to an EMT. However, this study provides clear evidence for E-cadherin loss promoting a reduction in cell-substrate adhesion and causing significant disruption to the normal organization of the microtubule and actin cytoskeletons.

### Availability of supporting data

Supporting RNAseq data are available in the GEO database (http://www.ncbi.nlm.nih.gov/geo/query/acc.cgi?acc=GSE59317) under accession number: GSE59317.

## Electronic supplementary material

Additional file 1: Figure S1: Original immunoblot depicting the expression level of E-cadherin in MCF10A and MCF10A CDH1-/- isogenic cells with α-actin expression as the loading control. The other lanes depict E-cadherin and α-actin expression from AGS wildtype cells (E-cadherin null) and AGSTrec1.3 cells stably expressing E-cadherin under the control of a doxycylin inducible promoter. The data from the AGS and AGSTrec1.3 cells are not part of this study. (DOC 664 KB)

Additional file 2: Figure S2: Histogram representing the proportion of cells with the different number of nucleolus per nucleus. Nuceloli numbers were counted independently twice from five images taken for each cell type and the average taken. The means and standard deviations are represented in the table. (DOC 106 KB)

Additional file 3: Table S1: Gene Ontology analysis. (DOC 31 KB)

Additional file 4: Table S2: Expression profile of selected cell-cell adhesion genes. Genes with negligible expression are excluded. Fold change expression is relative to MCF10A wildtype. (DOC 66 KB)

Additional file 5: Table S3: Expression profile of selected focal adhesion and ECM genes. Genes with negligible expression are excluded. Fold change expression is relative to MCF10A wildtype. (DOC 122 KB)

Additional file 6: Table S4: Expression profile of selected EMT related genes. Genes with negligible expression are also excluded. Fold change expression is relative to MCF10A wildtype. (DOC 84 KB)

Additional file 7: Table S5: Normalised expression profile of selected EMT related genes in their respective replicates in the isogenic cell lines. (DOC 58 KB)
