# Supplementary material for: E-cadherin loss alters cytoskeletal organization and adhesion in non-malignant breast cells but is insufficient to induce an epithelial-mesenchymal transition
Source: BMC Cancer. 2014 Jul 30;14:552. doi: 10.1186/1471-2407-14-552 (PMC4131020; doi:10.1186/1471-2407-14-552)
Supplement: Supplementary file 1 — Additional file 1: Figure S1: Original immunoblot depicting the expression level of E-cadherin in MCF10A and MCF10A CDH1-/- isogenic cells with α-actin expression as the loading control. The other lanes depict E-cadherin and α-actin expression from AGS wildtype cells (E-cadherin null) and AGSTrec1.3 cells stably expressing E-cadherin under the control of a doxycylin inducible promoter. The data from the AGS and AGSTrec1.3 cells are not part of this study. (DOC 664 KB) [file 12885_2014_4745_MOESM1_ESM.doc]

Figure S1. Original immunoblot depicting the expression level of E-cadherin in MCF10A and MCF10A *CDH1-/-* isogenic cells with α-actin expression as the loading control. The other lanes depict E-cadherin and α-actin expression from AGS wildtype cells (E-cadherin null) and AGSTrec1.3 cells stably expressing E-cadherin under the control of a doxycylin inducible promoter. The data from the AGS and AGSTrec1.3 cells are not part of this study.
