# Supplementary material for: E-cadherin loss alters cytoskeletal organization and adhesion in non-malignant breast cells but is insufficient to induce an epithelial-mesenchymal transition
Source: BMC Cancer. 2014 Jul 30;14:552. doi: 10.1186/1471-2407-14-552 (PMC4131020; doi:10.1186/1471-2407-14-552)
Supplement: Supplementary file 2 — Additional file 2: Figure S2: Histogram representing the proportion of cells with the different number of nucleolus per nucleus. Nuceloli numbers were counted independently twice from five images taken for each cell type and the average taken. The means and standard deviations are represented in the table. (DOC 106 KB) [file 12885_2014_4745_MOESM2_ESM.doc]

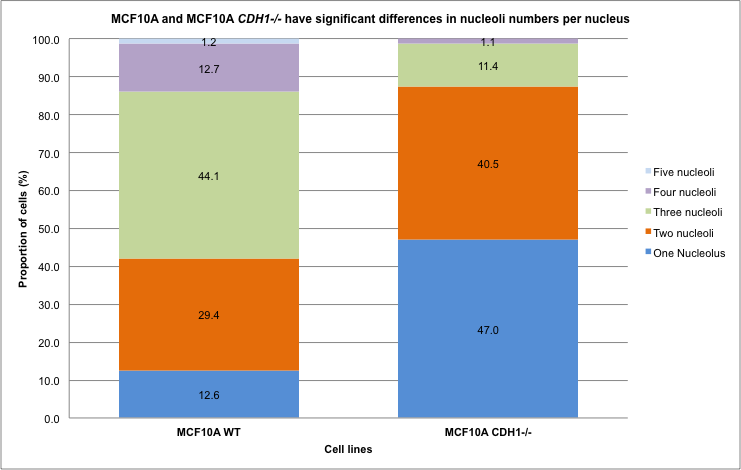


| Cell lines | Proportion of cells in percentage (mean ± standard deviation) | | | | |
| --- | --- | --- | --- | --- | --- |
|  | One nucleolus | Two nucleoli | Three nucleoli | Four nucleoli | Five nucleoli |
| MCF10A | 12.6 ± 3.9 | 29.4 ± 4.0 | 44.1 ± 11.5 | 12.7 ± 6.3 | 1.2 ± 0.5 |
| MCF10A *CDH1-/-* | 47.0 ± 5.9 | 40.5 ± 4.2 | 11.4 ± 3.4 | 1.1 ± 1.0 | 0 |

Figure S2. Histogram representing the proportion of cells with the different number of nucleolus per nucleus. Nuceloli numbers were counted independently twice from five images taken for each cell type and the average taken. The means and standard deviations are represented in the table.
