# Supplementary material for: E-cadherin loss alters cytoskeletal organization and adhesion in non-malignant breast cells but is insufficient to induce an epithelial-mesenchymal transition
Source: BMC Cancer. 2014 Jul 30;14:552. doi: 10.1186/1471-2407-14-552 (PMC4131020; doi:10.1186/1471-2407-14-552)
Supplement: Supplementary file 3 — Additional file 3: Table S1: Gene Ontology analysis. (DOC 31 KB) [file 12885_2014_4745_MOESM3_ESM.doc]

Table S1: Gene Ontology analysis

| GO Annotation | Number of differentially regulated genes | Bayes factor |
| --- | --- | --- |
| GO:0009653 [3]: morphogenesis | 154 | 34.1 |
| GO:0009887 [4]: organogenesis | 130 | 31.5 |
| GO:0048513 [3]: organ development | 130 | 31.5 |
| GO:0007275 [2]: development | 205 | 30.3 |
| GO:0008152 [3]: metabolism | 355 | 28.2 |
| GO:0044237 [4]: cellular metabolism | 329 | 27.8 |
| GO:0007154 [3]: cell communication | 326 | 24.0 |
| GO:0044238 [4]: primary metabolism | 321 | 22.1 |
| GO:0007155 [4]: cell adhesion | 86 | 21.3 |
| GO:0009888 [5]: histogenesis | 30 | 18.5 |
