# Supplementary material for: E-cadherin loss alters cytoskeletal organization and adhesion in non-malignant breast cells but is insufficient to induce an epithelial-mesenchymal transition
Source: BMC Cancer. 2014 Jul 30;14:552. doi: 10.1186/1471-2407-14-552 (PMC4131020; doi:10.1186/1471-2407-14-552)
Supplement: Supplementary file 4 — Additional file 4: Table S2: Expression profile of selected cell-cell adhesion genes. Genes with negligible expression are excluded. Fold change expression is relative to MCF10A wildtype. (DOC 66 KB) [file 12885_2014_4745_MOESM4_ESM.doc]

Table S2: Expression profile of selected cell-cell adhesion genes. Genes with negligible expression are excluded. Fold change expression is relative to MCF10A wildtype.

|  | Gene name | Fold Change | Adjusted P Value |
| --- | --- | --- | --- |
| *Tight junctions:* |  |  |  |
| Claudin 1 | *CLDN1* | 3.08 | 3.45E-05 |
| Claudin 4 | *CLDN4* | 3.78 | 4.87E-05 |
| Claudin 7 | *CLDN7* | 2.73 | 1.51E-05 |
| Claudin 12 | *CLDN12* | 1.13 | 1.71E-01 |
| Claudin 15 | *CLDN15* | -2.02 | 1.48E-02 |
| Claudin 22 | *CLDN22* | 1.05 | 7.39E-01 |
| Claudin 23 | *CLDN23* | 1.84 | 6.12E-02 |
| Occludin | *OCLN* | 2.87 | 2.27E-05 |
| Junctional adhesion molecule 1 | *F11R* | 1.29 | 2.04E-04 |
| Junctional adhesion molecule 3 | *JAM3* | -1.28 | 4.41E-03 |
| Cingulin | *CGN* | 2.99 | 1.80E-04 |
| Tight junction protein 1 | *TJP1* | -1.20 | 1.54E-01 |
| Tight junction protein 2 | *TJP2* | 1.12 | 5.46E-02 |
| Tight junction protein 3 | *TJP3* | 1.87 | 4.69E-04 |
| *Adherens junction:* |  |  |  |
| (E-cadherin) | *(CDH1)* | -10.00 | 9.10E-06 |
| N-cadherin | *CDH2* | -2.18 | 2.37E-04 |
| P-cadherin | *CDH3* | 1.77 | 1.51E-05 |
| R-cadherin | *CDH4* | -2.87 | 7.79E-04 |
| Cadherin 13 | *CDH13* | -1.23 | 7.18E-02 |
| Cadherin 16 | *CDH16* | 3.79 | 6.73E-04 |
| Cadherin 24 | *CDH24* | -1.41 | 3.13E-02 |
| Nectin-1 | *PVRL1* | 1.36 | 4.48E-04 |
| Nectin-2 | *PVRL2* | 1.45 | 5.87E-04 |
| Nectin-3 | *PVRL3* | -1.49 | 6.02E-03 |
| Nectin-4 | *PVRL4* | 2.19 | 1.01E-04 |
| α-catenin | *CTNNA1* | 1.12 | 1.66E-02 |
| β-catenin | *CTNNB1* | 1.03 | 4.20E-01 |
| p120-catenin | *CTNND1* | 1.19 | 2.71E-02 |
| Vinculin | *VCL* | -1.11 | 4.20E-01 |
| α-actinin1 | *ACTN1* | 1.07 | 3.03E-01 |
| α-actinin4 | *ACTN4* | 1.02 | 4.98E-01 |
| Formin | *FMN1* | 1.32 | 2.73E-01 |
| Cortactin | *CTTN* | 1.06 | 4.03E-01 |
| Afadin | *MLLT4* | 1.19 | 2.63E-01 |
| *Desmosome:* |  |  |  |
| Desmoglein 2 | *DSG2* | -1.18 | 4.90E-02 |
| Desmoglein 3 | *DSG3* | 1.66 | 1.24E-04 |
| Desmoglein 4 | *DSG4* | 3.03 | 9.73E-04 |
| Desmocollins2 | *DSC2* | 2.17 | 2.87E-05 |
| Desmocollins3 | *DSC3* | 1.45 | 3.01E-03 |
| *Gap Junction:* |  |  |  |
| Connexin46 | *GJA3* | -1.72 | 2.63E-03 |
| Connexin40 | *GJA5* | 2.10 | 1.13E-02 |
| Connexin26 | *GJB2* | 3.91 | 3.88E-06 |
| Connexin31 | *GJB3* | 1.55 | 1.46E-04 |
| Connexin30.3 | *GJB4* | 3.24 | 7.10E-04 |
| Connexin31.1 | *GJB5* | 1.66 | 3.28E-04 |
| Connexin45 | *GJC1* | -1.22 | 1.30E-01 |
| Connexin47 | *GJC2* | 1.71 | 2.19E-02 |
