# Supplementary material for: E-cadherin loss alters cytoskeletal organization and adhesion in non-malignant breast cells but is insufficient to induce an epithelial-mesenchymal transition
Source: BMC Cancer. 2014 Jul 30;14:552. doi: 10.1186/1471-2407-14-552 (PMC4131020; doi:10.1186/1471-2407-14-552)
Supplement: Supplementary file 5 — Additional file 5: Table S3: Expression profile of selected focal adhesion and ECM genes. Genes with negligible expression are excluded. Fold change expression is relative to MCF10A wildtype. (DOC 122 KB) [file 12885_2014_4745_MOESM5_ESM.doc]

Table S3. Expression profile of selected focal adhesion and ECM genes. Genes with negligible expression are excluded. Fold change expression is relative to MCF10A wildtype.

|  | *Gene name* | Fold Change | Adjusted P- value |
| --- | --- | --- | --- |
| *Extracellular Matrix:* |  |  |  |
| Collagen Type I, Alpha 1 | *COL1A1* | -1.40 | 1.36E-01 |
| Collagen Type II, Alpha 1 | *COL2A1* | 5.26 | 3.22E-04 |
| Collagen Type IV, Alpha 1 | *COL4A1* | -2.62 | 3.45E-05 |
| Collagen Type IV, Alpha 2 | *COL4A2* | -2.45 | 4.88E-05 |
| Collagen Type IV, Alpha 4 | *COL4A4* | -2.88 | 2.04E-02 |
| Collagen Type IV, Alpha 5 | *COL4A5* | -1.54 | 4.70E-03 |
| Collagen Type IV, Alpha 6 | *COL4A6* | -1.38 | 1.23E-02 |
| Collagen Type V, Alpha 2 | *COL5A2* | -1.01 | 8.42E-01 |
| Collagen Type V, Alpha 3 | *COL5A3* | 3.57 | 1.07E-03 |
| Collagen Type VI, Alpha 1 | *COL6A1* | 1.61 | 1.18E-03 |
| Collagen Type VI, Alpha 3 | *COL6A3* | -3.46 | 2.86E-03 |
| Collagen Type VII, Alpha 1 | *COL7A1* | 1.99 | 3.11E-03 |
| Collagen Type VIII, Alpha 1 | *COL8A1* | -6.34 | 1.12E-05 |
| Collagen Type XII, Alpha 1 | *COL12A1* | -4.31 | 4.48E-04 |
| Collagen Type XIII, Alpha 1 | *COL13A1* | 6.12 | 1.07E-04 |
| Collagen Type XVI, Alpha 1 | *COL16A1* | -1.54 | 8.52E-03 |
| Collagen Type XVII, Alpha 1 | *COL17A1* | 1.12 | 2.33E-01 |
| Collagen Type XVIII, Alpha 1 | *COL18A1* | -2.44 | 4.07E-04 |
| Collagen Type XXII, Alpha 1 | *COL22A1* | 1.35 | 1.78E-01 |
| Collagen Type XXVII, Alpha 1 | *COL27A1* | -6.28 | 7.07E-04 |
| Collagen Type XXVIII, Alpha 1 | *COL28A1* | -34.11 | 4.93E-03 |
| Laminin Alpha 1 | *LAMA1* | -2.49 | 8.51E-04 |
| Laminin Alpha 2 | *LAMA2* | 2.30 | 4.77E-04 |
| Laminin Alpha 3 | *LAMA3* | 1.15 | 3.58E-01 |
| Laminin Alpha 5 | *LAMA5* | -1.61 | 7.29E-03 |
| Laminin Beta 1 | *LAMB1* | -1.34 | 3.62E-03 |
| Laminin Beta 2 | *LAMB2* | -1.27 | 4.97E-02 |
| Laminin Beta 3 | *LAMB3* | 1.21 | 1.47E-02 |
| Laminin Gamma 1 | *LAMC1* | -1.58 | 4.62E-03 |
| Laminin Gamma 2 | *LAMC2* | 1.44 | 1.22E-03 |
| Fibronectin 1 | *FN1* | -7.24 | 1.78E-04 |
| Vitronectin | *VTN* | -1.33 | 6.07E-01 |
| *Focal adhesion:* |  |  |  |
| Integrin Alpha 1 | *ITGA1* | -2.81 | 6.37E-04 |
| Integrin Alpha 2 | *ITGA2* | 1.09 | 5.04E-01 |
| Integrin Alpha 3 | *ITGA3* | -1.08 | 3.63E-01 |
| Integrin Alpha 4 | *ITGA4* | -1.72 | 2.67E-02 |
| Integrin Alpha 5 | *ITGA5* | -1.19 | 8.75E-03 |
| Integrin Alpha 6 | *ITGA6* | 1.22 | 1.65E-02 |
| Integrin Alpha 10 | *ITGA10* | 2.04 | 6.92E-04 |
| Integrin Alpha E | *ITGAE* | 1.04 | 5.18E-01 |
| Integrin Alpha V | *ITGAV* | -1.40 | 2.11E-02 |
| Integrin Beta 1 | *ITGB1* | -1.38 | 2.91E-04 |
| Integrin Beta 2 | *ITGB2* | -1.65 | 4.09E-04 |
| Integrin Beta 4 | *ITGB4* | 1.14 | 1.91E-01 |
| Integrin Beta 5 | *ITGB5* | 1.01 | 6.44E-01 |
| Integrin Beta 6 | *ITGB6* | 1.31 | 1.40E-02 |
| Integrin Beta 7 | *ITGB7* | 1.47 | 1.76E-01 |
| Integrin Beta 8 | *ITGB8* | 1.14 | 4.20E-01 |
| Intercellular Adhesion Molecule 1 | *ICAM1* | -3.62 | 8.31E-03 |
| Intercellular Adhesion Molecule 3 | *ICAM3* | 1.10 | 3.30E-01 |
| Intercellular Adhesion Molecule 5 | *ICAM5* | -1.81 | 6.11E-02 |
| Talin 1 | *TLN1* | -1.54 | 1.48E-02 |
| Talin 2 | *TLN2* | -1.96 | 5.72E-03 |
| Tensin 1 | *tns1* | -5.53 | 7.34E-05 |
| Tensin 3 | *tns3* | -2.27 | 2.77E-04 |
| Vinculin | *VCL* | -1.11 | 4.20E-01 |
| Actinin Alpha 1 | *ACTN1* | 1.02 | 4.98E-01 |
| Actinin Alpha 4 | *ACTN4* | 1.07 | 3.03E-01 |
| Filamin A Alpha | *FLNA* | -1.23 | 1.21E-01 |
| Filamin B Beta | *FLNB* | -1.06 | 6.13E-01 |
| Parvin beta | *parvb* | 1.38 | 2.51E-03 |
| Palladin | *palld* | 1.16 | 1.19E-01 |
| Paxillin | *pxn* | -1.12 | 1.65E-02 |
| Profilin 1 | *pfn1* | 1.09 | 4.20E-01 |
| Profilin 2 | *pfn2* | 1.07 | 1.20E-01 |
| Profilin 3 | *pfn3* | 1.71 | 4.59E-01 |
| Deleted in liver cancer 1 | *DLC1* | -2.20 | 1.19E-03 |
| Ezrin | *ezr* | 1.21 | 1.22E-03 |
| Moesin | *msn* | -1.11 | 3.06E-02 |
| Radixin | *rdx* | -1.13 | 1.04E-01 |
| Enabled homolog | *enah* | -1.23 | 4.92E-03 |
| Zyxin | *ZYX* | 1.05 | 1.67E-01 |
| c-src Tyrosine Kinase | *csk* | 1.04 | 4.20E-01 |
| v-src avian sarcoma viral oncogene homolog | *src* | -1.25 | 2.29E-02 |
| v-crk avian sarcoma virus C10 oncogene homolog | *crk* | 1.04 | 3.13E-01 |
| Protein Tyrosine Kinase 2 | *ptk2* | 1.14 | 1.48E-02 |
| Protein tyrosine kinase 2 beta | *Ptk2b* | -1.01 | 7.94E-01 |
| Integrin-linked kinase | *ILK* | 1.04 | 4.20E-01 |
| Protein tyrosine phosphatase non receptor type 1 | *PTPN1* | -1.08 | 3.49E-01 |
| Ras-related C3 botulinum toxin substrate 1 | *rac1* | 1.14 | 2.29E-02 |
| Ras homolog family member A | *RHOA* | 1.09 | 1.89E-02 |
| Ras homolog family member B | *RHOB* | 1.42 | 2.70E-02 |
| Ras homolog family member C | *RHOC* | 1.44 | 8.34E-04 |
| Rho-Associated, Coiled-Coil-Containing Protein Kinase 1 | *ROCK1* | -1.28 | 4.28E-02 |
| Rho-Associated, Coiled-Coil-Containing Protein Kinase 2 | *ROCK2* | -1.26 | 1.08E-01 |
| actin related protein 2/3 complex subunit 1A | *ARPC1A* | 1.07 | 2.52E-01 |
| actin related protein 2/3 complex subunit 1B | *ARPC1B* | 1.22 | 9.59E-03 |
| actin related protein 2/3 complex subunit 2 | *ARPC2* | 1.19 | 3.38E-03 |
| actin related protein 2/3 complex subunit 3 | *ARPC3* | 1.16 | 2.34E-02 |
| actin related protein 2/3 complex subunit 4 | *ARPC4* | 1.18 | 3.72E-02 |
| actin related protein 2/3 complex, subunit 5 | *ARPC5* | 1.07 | 2.07E-01 |
| Vasodilator-stimulated phosphoprotein | *VASP* | 1.38 | 4.17E-04 |
| c-abl Oncogene 1 | *Abl1* | -1.37 | 1.22E-03 |
| Caveolin 1 | *cav1* | -1.01 | 5.85E-01 |
| Cell division cycle 42 | *cdc42* | 1.14 | 5.00E-02 |
| Breast cancer anti- estrogen resistance 1 (p130Cas) | *bcar1* | -1.08 | 2.51E-01 |
| LIM and senescent cell antigen-like domains 1 | *lims1* | -1.10 | 8.74E-02 |
| Ninein | *NIN* | -1.36 | 6.13E-02 |
| Kinesin family member C3 | *KIFC3* | -1.61 | 1.04E-04 |
| Dynamin2 | *DNM2* | -1.04 | 4.20E-01 |
| Testis derived transcript | *tes* | 1.06 | 3.06E-01 |
| P21 protein-activated kinase 1 | *pak1* | 1.02 | 6.19E-01 |
| P21 protein-activated kinase 2 | *pak2* | -1.22 | 6.47E-03 |
| P21 protein-activated kinase 3 | *pak3* | -1.42 | 1.74E-01 |
| P21 protein-activated kinase 4 | *pak4* | -1.03 | 4.60E-01 |
| P21 protein-activated kinase 6 | *pak6* | 1.31 | 7.64E-03 |
| Protein kinase C alpha | *prkca* | 1.28 | 2.34E-01 |
| Protein kinase C zeta | *prkcz* | 1.01 | 8.80E-01 |
| Protein kinase C eta | *prkch* | -1.11 | 1.28E-01 |
| Syndecan 4 | *sdc4* | 1.27 | 6.89E-03 |
| Syndecan 1 | *sdc1* | 1.63 | 3.11E-05 |
| Syndecan 3 | *sdc3* | -1.90 | 2.05E-04 |
| Calpain 2 large subunit | *capn2* | 1.29 | 1.09E-04 |
| Gelsolin | *gsn* | 1.95 | 2.71E-05 |
| Sorbin and SH3 domain containing 3 | *SORBS3* | 1.14 | 8.33E-03 |
| Syntrophin alpha 1 | *snta1* | 1.24 | 3.03E-01 |
| Syntrophin alpha 2 | *sntb2* | -1.47 | 2.25E-02 |
| Dystrophin | *dmd* | 1.13 | 4.77E-02 |
| Plastin 3 | *PLS3* | 1.24 | 4.23E-04 |
| Utrophin | *utrn* | -2.41 | 1.13E-02 |
| Dystroglycan 1 | *DAG1* | 1.13 | 2.49E-02 |
| Phosphatase and tensin homolog | *pten* | 1.06 | 4.20E-01 |
