# Supplementary material for: E-cadherin loss alters cytoskeletal organization and adhesion in non-malignant breast cells but is insufficient to induce an epithelial-mesenchymal transition
Source: BMC Cancer. 2014 Jul 30;14:552. doi: 10.1186/1471-2407-14-552 (PMC4131020; doi:10.1186/1471-2407-14-552)
Supplement: Supplementary file 6 — Additional file 6: Table S4: Expression profile of selected EMT related genes. Genes with negligible expression are also excluded. Fold change expression is relative to MCF10A wildtype. (DOC 84 KB) [file 12885_2014_4745_MOESM6_ESM.doc]

Table S4: Expression profile of selected EMT related genes. Genes with negligible expression are also excluded. Fold change expression is relative to MCF10A wildtype.

|  | Gene name | Fold Change | Adjusted P-Value |
| --- | --- | --- | --- |
| Keratin 8 | *KRT8* | 1.42 | 7.67E-04 |
| Keratin 9 | *KRT9* | 2.48 | 2.89E-02 |
| Keratin 18 | *KRT18* | 1.36 | 3.86E-04 |
| Claudin 1 | *CLDN1* | 3.08 | 3.45E-05 |
| Occludin | *OCLN* | 2.87 | 2.27E-05 |
| Tight junction protein 3 | *TJP3* | 1.87 | 4.69E-04 |
| Cingulin | *CGN* | 2.99 | 1.80E-04 |
| Desmoplakin | *DSP* | 1.00 | 9.87E-01 |
| Syndecan 1 | *SDC1* | 1.63 | 3.11E-05 |
| Syndecan 2 | *SDC2* | 1.28 | 4.20E-01 |
| Integrin Alpha 5 | *ITGA5* | -1.19 | 8.75E-03 |
| Integrin Beta 1 | *ITGB1* | -1.38 | 2.91E-04 |
| Integrin Alpha V | *ITGAV* | -1.40 | 2.11E-02 |
| Integrin Beta 6 | *ITGB6* | 1.31 | 1.40E-02 |
| N-cadherin | *CDH2* | -2.18 | 2.37E-04 |
| OB-cadherin (Osteoblast) | *CDH11* | 1.40 | 3.11E-01 |
| Vimentin | *VIM* | 1.09 | 6.12E-02 |
| Fibronectin 1 | *FN1* | -7.24 | 1.78E-04 |
| S100 calcium binding protein A1 | *S100A1* | -1.11 | 4.93E-01 |
| S100 calcium binding protein A2 | *S100A2* | 1.49 | 1.02E-02 |
| S100 calcium binding protein A3 | *S100A3* | 1.35 | 7.82E-03 |
| S100 calcium binding protein A4 | *S100A4* | -1.07 | 4.20E-01 |
| S100 calcium binding protein A6 | *S100A6* | 1.41 | 3.62E-02 |
| S100 calcium binding protein A7 | *S100A7* | 7.11 | 8.3E-04 |
| S100 calcium binding protein A8 | *S100A8* | 4.11 | 1.11E-04 |
| S100 calcium binding protein A9 | *S100A9* | 2.41 | 1.19E-03 |
| S100 calcium binding protein A10 | *S100A10* | 1.61 | 1.11E-03 |
| S100 calcium binding protein A11 | *S100A11* | 1.65 | 2.60E-03 |
| S100 calcium binding protein A23 | *S100A13* | 1.25 | 1.65E-03 |
| S100 calcium binding protein A14 | *S100A14* | 2.01 | 6.73E-04 |
| S100 calcium binding protein A16 | *S100A16* | 1.40 | 1.80E-03 |
| Matrix metallopeptidase 2 | *MMP2* | -1.21 | 8.78E-02 |
| Matrix metallopeptidase 9 | *MMP9* | 3.31 | 9.08E-04 |
| Matrix metallopeptidase 14 | *MMP14* | 1.49 | 4.48E-05 |
| Matrix metallopeptidase 15 | *MMP15* | 1.21 | 1.18E-01 |
| Matrix metallopeptidase 16 | *MMP16* | -2.18 | 1.10E-02 |
| Matrix metallopeptidase 17 | *MMP17* | 2.31 | 6.52E-04 |
| Matrix metallopeptidase 19 | *MMP19* | -1.50 | 2.51E-02 |
| Matrix metallopeptidase 28 | *MMP28* | 1.97 | 3.68E-05 |
| Laminin alpha 5 | *LAMA5* | -1.61 | 7.29E-03 |
| Collagen Type I, alpha 1 | *COL1A1* | -1.40 | 1.36E-01 |
| Collagen Type II, alpha 1 | *COL2A1* | 5.26 | 3.22E-04 |
| Catenin beta 1 | *CTNNB1* | 1.03 | 4.20E-01 |
| Actin alpha 2 | *ACTA2* | 1.01 | 9.12E-01 |
| Wingless-type MMTV integration site family member 10A | *WNT10A* | 1.05 | 7.81E-01 |
| Wingless-type MMTV integration site family member 7A | *WNT7B* | 1.03 | 8.24E-01 |
| Snail family zinc finger 1 | *SNAI1* | -2.49 | 1.38E-02 |
| Snail family zinc finger 2 | *SNAI2* | 1.02 | 5.83E-01 |
| Twist basic helix-loop-helix transcription factor 1 | *TWIST1* | -1.14 | 3.58E-01 |
| Twist basic helix-loop-helix transcription factor 2 | *TWIST2* | -1.15 | 4.20E-01 |
| Zinc finger E-box binding homeobox 1 | *ZEB1* | -1.68 | 1.23E-02 |
| Zinc finger E-box binding homeobox 2 | *ZEB2* | -7.28 | 5.02E-05 |
| Forkhead box C2 | *FOXC2* | -1.26 | 2.38E-02 |
| Lymphoid enhancer-binding factor 1 | *LEF1* | -1.76 | 1.02E-02 |
| Transforming growth factor beta 1 | *TGFB1* | 1.14 | 9.14E-02 |
| Nuclear factor of kappa light polypeptide gene enhancer in B-cells 1 | *NFKB1* | -1.25 | 2.25E-02 |
| CD44 molecule | *CD44* | 1.48 | 9.67E-05 |
| TIMP metallopeptidase inhibitor 1 | *TIMP1* | -1.18 | 1.53E-01 |
| TIMP metallopeptidase inhibitor 2 | *TIMP2* | -1.21 | 5.12E-03 |
| TIMP metallopeptidase inhibitor 3 | *TIMP3* | -1.20 | 1.01E-03 |
| Kallikrein 1 | *KLK1* | 2.50 | 5.82E-03 |
| Kallikrein related peptidase 5 | *KLK5* | 4.54 | 3.71E-05 |
| Kallikrein related peptidase 6 | *KLK6* | 4.13 | 2.60E-03 |
| Kallikrein related peptidase 7 | *KLK7* | 3.73 | 1.51E-05 |
| Kallikrein related peptidase 8 | *KLK8* | 5.20 | 7.51E-05 |
| Kallikrein related peptidase 9 | *KLK9* | 4.60 | 3.45E-05 |
| Kallikrein related peptidase 10 | *KLK10* | 4.00 | 2.27E-05 |
| Kallikrein related peptidase 11 | *KLK11* | 8.41 | 2.85E-04 |
| Kallikrein related peptidase 13 | *KLK13* | 4.00 | 2.03E-03 |
| Kallikrein related peptidase 14 | *KLK14* | 3.05 | 1.29E-03 |
