# Supplementary material for: E-cadherin loss alters cytoskeletal organization and adhesion in non-malignant breast cells but is insufficient to induce an epithelial-mesenchymal transition
Source: BMC Cancer. 2014 Jul 30;14:552. doi: 10.1186/1471-2407-14-552 (PMC4131020; doi:10.1186/1471-2407-14-552)
Supplement: Supplementary file 7 — Additional file 7: Table S5: Normalised expression profile of selected EMT related genes in their respective replicates in the isogenic cell lines. (DOC 58 KB) [file 12885_2014_4745_MOESM7_ESM.doc]

Table S5: Normalised expression profile of selected EMT related genes in their respective replicates in the isogenic cell lines.

|  | MCF10A *CDH1-/-*_rep1 | MCF10A *CDH1-/-*_rep2 | MCF10A_rep1 | MCF10A_rep2 |
| --- | --- | --- | --- | --- |
| KRT8 | 385.3 | 361.4 | 261.6 | 264.4 |
| KRT9 | 1.8 | 1.3 | 0.8 | 0.5 |
| KRT18 | 462.8 | 451.0 | 333.4 | 336.9 |
| CLDN1 | 166.7 | 155.2 | 54.6 | 50.1 |
| OCLN | 79.4 | 75.8 | 27.8 | 26.3 |
| TJP3 | 18.7 | 18.9 | 10.2 | 10.0 |
| CGN | 19.0 | 21.7 | 6.8 | 6.9 |
| DSP | 772.5 | 1099.1 | 904.2 | 942.9 |
| SDC1 | 588.9 | 570.9 | 357.2 | 352.6 |
| CDH2 | 10.4 | 9.1 | 20.5 | 21.6 |
| CDH11 | 0.9 | 0.7 | 0.5 | 0.6 |
| VIM | 638.4 | 615.4 | 577.6 | 574.2 |
| FN1 | 34.7 | 53.1 | 308.9 | 317.6 |
| CTNNB1 | 309.0 | 309.6 | 307.4 | 295.9 |
| ACTA2 | 8.5 | 8.2 | 8.0 | 8.5 |
| ITGA5 | 155.0 | 164.8 | 190.1 | 189.9 |
| ITGAV | 123.2 | 136.4 | 191.4 | 172.4 |
| SNAI1 | 0.9 | 0.6 | 2.0 | 1.8 |
| SNAI2 | 73.5 | 76.8 | 74.1 | 73.0 |
| TWIST1 | 15.2 | 12.6 | 15.0 | 16.3 |
| TWIST2 | 12.0 | 9.4 | 12.2 | 12.0 |
| ZEB1 | 4.0 | 5.1 | 7.8 | 7.6 |
| ZEB2 | 2.3 | 2.9 | 17.9 | 19.8 |
| AKT2 | 129.8 | 137.9 | 145.5 | 146.2 |
| MMP1 | 0.9 | 1.4 | 0.1 | 0.3 |
| MMP9 | 5.3 | 4.5 | 1.7 | 1.3 |
| MMP14 | 196.9 | 195.1 | 133.2 | 130.5 |
